# Zoledronate/Anti-VEGF Neutralizing Antibody Combination Administration Increases Osteal Macrophages in a Murine Model of MRONJ Stage 0-like Lesions

**DOI:** 10.3390/jcm12051914

**Published:** 2023-02-28

**Authors:** Haruka Kaneko, Shinichiro Kuroshima, Ryohei Kozutsumi, Farah A. Al-Omari, Hiroki Hayano, Kazunori Nakajima, Takashi Sawase

**Affiliations:** Department of Applied Prosthodontics, Graduate School of Biomedical Sciences, Nagasaki University, Nagasaki 852-8588, Japan

**Keywords:** osteal macrophage, angiogenesis inhibitor, zoledronate, wound healing, tooth extraction

## Abstract

The pathophysiology, pathogenesis, histopathology, and immunopathology of medication-related osteonecrosis of the jaw (MRONJ) Stage 0 remain unclear, although 50% of MRONJ Stage 0 cases could progress to higher stages. The aim of this study was to investigate the effects of zoledronate (Zol) and anti-vascular endothelial cell growth factor A (VEGFA) neutralizing antibody (Vab) administration on polarization shifting of macrophage subsets in tooth extraction sockets by creating a murine model of MRONJ Stage 0-like lesions. Eight-week-old, female C57BL/6J mice were randomly divided into 4 groups: Zol, Vab, Zol/Vab combination, and vehicle control (VC). Subcutaneous Zol and intraperitoneal Vab administration were performed for 5 weeks with extraction of both maxillary first molars 3 weeks after drug administration. Euthanasia was conducted 2 weeks after tooth extraction. Maxillae, tibiae, femora, tongues, and sera were collected. Structural, histological, immunohistochemical, and biochemical analyses were comprehensively performed. Tooth extraction sites appeared to be completely healed in all groups. However, osseous healing and soft tissue healing of tooth extraction sites were quite different. The Zol/Vab combination significantly induced abnormal epithelial healing, and delayed connective tissue healing due to decreased rete ridge length and thickness of the stratum granulosum and due to decreased collagen production, respectively. Moreover, Zol/Vab significantly increased necrotic bone area with increased numbers of empty lacunae compared with Vab and VC. Most interestingly, Zol/Vab significantly increased the number of CD169^+^ osteal macrophages (osteomacs) in the bone marrow and decreased F4/80+ macrophages, with a slightly increased ratio of F4/80^+^CD38^+^ M1 macrophages compared to VC. These findings are the first to provide new evidence of the involvement of osteal macrophages in the immunopathology of MRONJ Stage 0-like lesions.

## 1. Introduction

Angiogenesis inhibitors, such as the anti-vascular endothelial growth factor (VEGF) monoclonal antibody bevacizumab, have been used worldwide to treat patients with malignancy [1]. Malignant tumors have clearly been increasing up to more than twice worldwide from 1990 to 2019 [2]. Medication-related osteonecrosis of the jaw (MRONJ), which is rare but shows potentially severe impaired healing of hard and soft tissues in the oral and maxillofacial regions, has been reported to be induced by antiresorptive agents such as bisphosphonates and denosumab, or in combination with angiogenesis inhibitors [3].

The first 36 cases of bisphosphonate-related osteonecrosis of the jaw (BRONJ) were referred to as “avascular necrosis of the jaw” [4]. Angiogenesis inhibitor use has been reported to be one of the risk factors for developing MRONJ [3,5]. The frequency of MRONJ induced by angiogenesis inhibitor monotherapy in advanced breast cancer patients has been reported to range from 0.3% to 0.4%, although angiogenesis inhibitor monotherapy was excluded from the new definition of MRONJ in 2022 by the Association of American Oral & Maxillofacial Surgeons (AAOMS) [3,6]. Interestingly, the prevalence is markedly higher with the combined use of bevacizumab and bisphosphonate than with angiogenesis inhibitor monotherapy (0.9% to 2.4%) [7].

Osteal macrophages, known as osteomacs [8], have been observed close to bone surfaces and comprise a specific niche in the bone marrow by linking with osteoblasts, megakaryocytes, and other cells [8,9]. Moreover, osteal macrophages have been shown to control bone homeostasis and osseous wound healing of injured long bones [10,11,12,13]. However, there is limited evidence regarding osteal macrophages in oral and maxillofacial regions, although polarization shifting of macrophages in the connective tissue has been demonstrated to occur in human MRONJ and rodent MRONJ-like lesions [14,15,16,17]. Consequently, the effects of antiresorptive drugs and anti-VEGFA antibody combination administration on osteal macrophages in tooth extraction sites have not been elucidated.

MRONJ is clinically classified as Stage 0 to 3 according to the severity of clinical symptoms and radiographic findings. This classification has been agreed upon by many MRONJ committees, including in the USA [3], Germany [18], Korea [19], and Japan [20]. Importantly, it has been reported that 50% of MRONJ Stage 0 cases without bone exposure progressed to higher stages of MRONJ [21]. Moreover, MRONJ Stage 0 accounts for about 30% of MRONJ cases [21], although the reasons or mechanisms behind why MRONJ Stage 0 progresses to higher stages have not been fully understood due to the limited animal models of MRONJ Stage 0-like lesions. More recently, we created a murine model of BRONJ Stage 0-like lesions induced by zoledronate (Zol) administration without bone exposure but with increased necrotic bone and abnormal soft tissue wound healing of tooth extraction sockets in a Zol-dependent manner [22]. Moreover, previously, we demonstrated that Vab administration impaired hard tissue socket healing, although no bone exposure was observed [23], similar to the findings of MRONJ Stage 0 in humans.

From the abovementioned scientific and clinical data, we hypothesized that alteration of macrophage subsets in the tooth extraction sockets predominantly occurs from MRONJ Stage 0, which is an early stage of MRONJ in the clinical setting. The aim of this study was to investigate the effects of Zol and anti-VEGFA antibody (Vab) administration on macrophage subsets in soft and hard tissues of tooth extraction sockets by creating a murine model of MRONJ Stage 0-like lesions.

## 2. Materials and Methods

### 2.1. Animals and Drugs Administered

**Figure 1:**
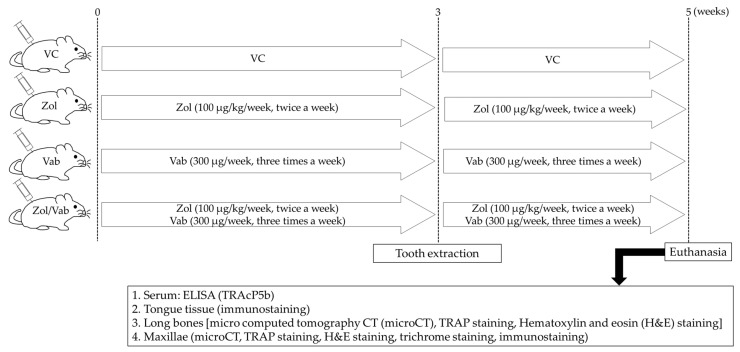
Experimental schedule. Zoledronate (Zol), anti-VEGF-A neutralizing antibody (Vab), and their combination (Zol/Vab) are administered for 5 weeks. Tooth extraction and euthanasia are carried out 3 and 5 weeks, respectively, after onset of drug administration.

Twenty-three, 8-week-old, female C57BL/6J mice were used. Bisphosphonate (zoledronate; Zol, Novartis, Stein, Switzerland) and anti-VEGFA neutralizing antibody (clone 2G11-2A05; BioLegend, San Diego, CA, USA) were purchased. Mice were randomly divided into 4 groups as follows: Zol monotherapy group (Zol), anti-VEGFA neutralizing antibody monotherapy group (Vab), Zol and anti-VEGFA neutralizing antibody combination therapy group (Zol/Vab), and control group (vehicle control: VC). Subcutaneous administration of Zol (100 µg/kg/week) and intraperitoneal administration of Vab (300 µg/week) were performed twice a week and every 2 days, respectively, for 5 weeks (*n* = 5 per each group). Both maxillary first molars were extracted 3 weeks after drug administration. Euthanasia was performed 2 weeks after tooth extraction (Figure 1). Animal care and experimental procedures were performed in accordance with the Guidelines for Animal Experimentation of Nagasaki University, with approval from the Ethics Committee for Animal Research. All animal experiments in this study were performed according to the ARRIVE Guidelines (https://arriveguidelines.org/resources/author-checklists, (accessed on 27 December 2022)). Three mice were excluded from this study due to fractures of roots of the extracted tooth (2 mice) or death during tooth extraction (1 mouse). All animal experiments excluding tooth extraction (S. K.) were performed by one operator (H. K.) to avoid potential confounding.

### 2.2. Evaluation of Gross Wound Healing of Tooth Extraction Sites

Gross wound healing of tooth extraction sockets was examined to evaluate the effects of the administered drugs on epithelial coverage of tooth extraction sockets. Occlusal photos were taken with higher resolution and magnification at euthanasia 2 weeks after tooth extraction. The number of open wounds (animal and extraction site levels) and wound perimeter and area were quantitatively analyzed.

### 2.3. Micro Computed Tomography (microCT)

To assess the effects of the administered drugs on bone architecture of maxillary and long bones, microCT was used (R_mCT2; Rigaku Co. Ltd., Tokyo, Japan). Twenty right maxillary and right tibial samples were fixed in 10% neutral buffered formalin (Muto Pure Chemicals Co. Ltd., Tokyo, Japan) for 24 h at 4 °C, followed by microCT with 20-μm voxels and tube voltage of 90 kV. The segmentations and reconstructions of maxillary and tibial bones were performed with TRI/3D-Bon (Ratoc System Engineering, Tokyo, Japan) [24,25].

### 2.4. Hematoxylin and Eosin Staining, Trichrome Staining, and Tartrate-Resistant Acid Phosphatase Staining

Twenty left maxillae, 20 right femora, and 20 tongues were fixed in 10% neutral buffered formalin at 4 °C for 24 h. Maxillary and femoral samples were decalcified by ethylenediaminetetraacetic acid (pH 7.3, FUJIFILM Wako Pure Chemical Co., Osaka, Japan) at 4 °C for 28 days. They were embedded in the paraffin and cut at a thickness of 5 µm. Hematoxylin and eosin (H&E) staining was conducted to visualize epithelium, connective tissue, necrotic bone, living bone, osteocytes, and empty lacunae in soft and hard tissues of tooth extraction sockets. Trichrome staining (HT15; Sigma-Aldrich, St. Louis, MO, USA) was performed to visualize collagen fibers in the connective tissue of tooth extraction sockets. Tartrate-resistant acid phosphatase (TRAP) staining was conducted to identify osteoclasts on the bone surface in tooth extraction sockets and femoral metaphyses. A light microscope (Axio Scope A1; Carl Zeiss, Gottingen, Germany) was used for taking images of all stained sections.

### 2.5. Immunofluorescent Staining

Immunofluorescent staining was conducted to identify blood vessels and macrophages (M1, M2, and osteal macrophages) in soft and hard tissues of tooth extraction sockets. Sections were prepared with deparaffinization, dehydration, antigen retrieval, and then blocking. Blood vessels in tongue and connective tissues of tooth extraction sockets were visualized using rat anti-mouse monoclonal antibody to CD31 at 1:100 (ab56299; Abcam, Cambridge, MA, USA) and the Alexa Fluor™ 546 goat anti-rat IgG (H+L) cross-adsorbed secondary antibody at 1:200 (A-11081; Invitrogen, Carlsbad, CA, USA). CD38 and CD163 were chosen as detection antibodies for M1 and M2 macrophages, respectively. Pretreated sections were incubated with goat F(ab) anti-mouse IgG H&L polyclonal antibody (ab6668; Abcam; 1:20) for 1 h to block endogenous mouse IgG in tissue sections. M1 macrophages were visualized using the cocktail of pan-macrophage marker rat anti-mouse monoclonal antibody to F4/80 at 1:25 (ab16911, Abcam) and mouse anti-human CD38 monoclonal antibody at 1:50 (sc-374650; Santa Cruz Biotechnology, Dallas, TX, USA), followed by the cocktail of the Alexa Fluor™ 546 goat anti-rat IgG (H+L) cross-adsorbed secondary antibody at 1:200 (A-11081, Invitrogen) and goat anti-mouse IgG (H&L) (FITC) (ab6785, Abcam) (both 1:200). M2 macrophages were visualized using the cocktail of rat anti-mouse monoclonal antibody to F4/80 at 1:25 (ab16911, Abcam) and mouse anti-rat CD163 antibody at 1:50 (sc-58965, Santa Cruz Biotechnology), followed by the cocktail of the Alexa Fluor 546 goat anti-rat IgG and goat anti-mouse IgG (H&L) (FITC) (ab6785, Abcam) (both 1:100). Moreover, osteal macrophages in the bone marrow of tooth extraction sites were visualized using rat anti-mouse monoclonal antibody to CD169 at 1:50 (ab53443, Abcam), followed by the Alexa Fluor™ 546 goat anti-rat IgG (H+L) cross-adsorbed secondary antibody at 1:200 (A-11081, Invitrogen). Finally, sections treated with first and secondary antibodies were mounted by VECTASHIELD Antifade Mounting Medium with DAPI (H-1200; Vector Laboratories, Burlingame, CA, USA). An immunofluorescent microscope (Zeiss) was used for taking images of all stained sections. Acquired images were semiautomatically and quantitatively analyzed with Zen2 software (Zeiss) and ImageJ [version 1.47; National Institutes of Health, Bethesda, MD, USA].

### 2.6. Evaluation of Drug Effects on Systemic Bones and Distribution of Blood Vessels in the Tongue

Systemic bone was examined to evaluate the effects of the drugs administered. Regions of interest (ROIs) of tibiae ranged from 200 to 2200 µm below the growth plate of the mesial metaphysis. Semiautomatic measurements of bone volume per tissue volume (BV/TV), trabecular number (Tb.N), trabecular thickness (Tb.Th), trabecular separation (Tb.Sp), and bone mineral density (BMD) were performed based on the guidelines for the evaluation of bone using microCT [26]. The number of osteoclasts on the femoral bone surface was counted from 200 to 2200 µm below the growth plate of the mesial metaphysis.

The distribution of blood vessels was also examined to evaluate the effects of the drugs administered. The number of CD31^+^ blood vessels was semiautomatically counted by averaging data of 2 randomly selected areas of interest (AOIs) (100 µm × 400 µm) in the connective tissue of tongues.

### 2.7. Evaluation of Osseous Wound Healing of Tooth Extraction Sockets

MicroCT analysis was used to evaluate bone architecture of tooth extraction sockets. H&E staining and TRAP staining were also used to assess bone architecture. The regions of interest (ROIs) of tooth extraction sockets ranged from the top of alveolar bone to the apex of mesial and two distal dental roots.

Bone fill in the extraction sockets (BV/TV), Tb.Th, Tb.N, Tb.Sp, and BMD in the ROIs were analyzed quantitatively. Bone area per tissue area [BA/TA (%)], the bone area with normal osteocytes in lacunae per total bone area [living bone (%)], the number of osteocytes in living bone [osteocyte number (#/mm^2^)], the bone area with more than 10 empty lacunae and pyknotic osteocytes per total bone tissue [necrotic bone (%)], and the number of empty lacunae in total bone [empty lacunae (#/mm^2^)] were also evaluated. The number of osteoclasts on the bone surface of AOIs [N.Oc/BS (#/mm)] was quantitatively counted.

### 2.8. Evaluation of Soft Tissue Healing of Tooth Extraction Sockets

Trichrome staining was performed for quantitative analysis of collagen production in the AOIs of connective tissue of tooth extraction sockets. Epithelial conditions including epithelial thickness, rete ridge length, and thicknesses of the stratum corneum, stratum granulosum, stratum spinosum, and stratum basale were quantitatively evaluated by average values with 5 different measurement sites of epithelium using H&E-stained sections. Moreover, immunostaining with CD31 antibody was also performed to investigate the distribution of blood vessels in the connective tissue of tooth extraction sockets.

### 2.9. Evaluation of Macrophages in the Tooth Extraction Socket

M1 and M2 macrophages in the connective tissue and osteal macrophages in bone marrow of tooth extraction sockets were evaluated. The numbers of F4/80^+^CD38^+^ cells and F4/80^+^CD163^+^ cells in the connective tissue of tooth extraction sockets were semiautomatically counted. The number of CD169^+^ cells in the bone marrow of tooth extraction sockets was also quantitatively measured.

### 2.10. Assessment of Serum TRAP Isoform 5b (TRAcP5b) by Enzyme-Linked Immunosorbent Assay (ELISA)

To examine the effects of the drugs administered on serum TRAcP5b, ELISA was conducted with the Mouse TRAPTM Assay (TRAcP5b ELISA) (DS-SBTR103; Immunodiagnostic Systems Ltd., Boldon, Tyne and Wear, UK). Cardiac puncture and centrifugation (2000 rpm for 30 min) were performed to collect fresh serum just after euthanasia. Acquired sera were then kept at −80 °C to just before use (*n* = 20). Serum levels of TRAcP5b were measured with a microplate reader at an absorbance of 405 nm (MultiSkan FC Advance; Thermo Scientific, Waltham, MA, USA). Mean values with duplicated data were used as measurement values.

### 2.11. Statistical Analysis

The Shapiro-Wilk test was conducted to evaluate normality. One-way analysis of variance (ANOVA) and the Kruskal-Wallis test were performed for parametric and non-parametric data, respectively. All data are expressed as means ± SEM. A *p*-value < 0.05 was considered significant. Systat 13.2 (Systat Software, Chicago, IL, USA) was used for all statistical analyses.

## 3. Results

### 3.1. Effects of Drugs on Systemic Conditions

To confirm the effects of Zol and Vab on systemic conditions, serum TRAcP5b levels, architecture of long bones, osteoclast distribution on the long bone surface, and distribution and surface area of blood vessels in the connective tissue of tooth extraction sockets were investigated.

**Figure 2:**
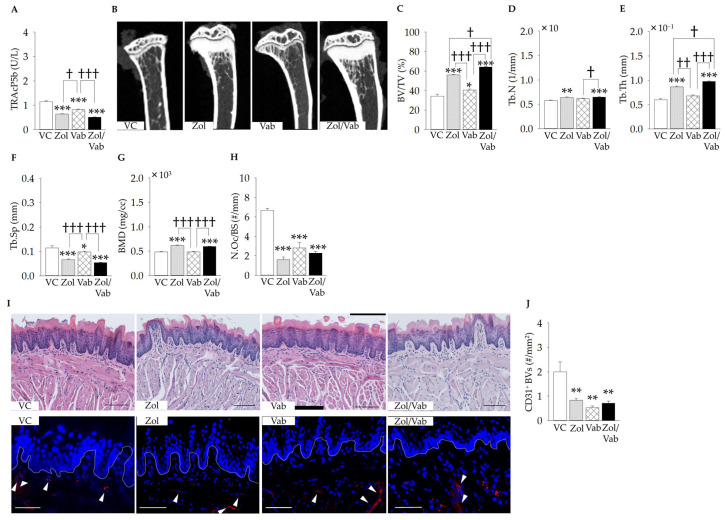
Effects of administered drugs on systemic conditions. (**A**) Serum TRAcP5b levels are significantly lower in Zol, Vab, and Zol/Vab vs. VC, and in Zol and Zol/Vab vs. Vab. (**B**) Representative micro computed tomography images of the tibial metaphysis. (**C**) Bone volume per tissue volume (BV/TV) is significantly larger in Zol/Vab vs. other comparison groups, whereas BV/TV is significantly larger in Zol and Vab vs. VC, and in Zol vs. Vab. (**D**) Trabecular number (Tb.N) is significantly increased in Zol/Vab vs. VC and Vab, and Zol vs. VC. (**E**) Trabecular thickness (Tb.Th) is significantly greater in Zol/Vab vs. other comparison groups, whereas Tb.Th is significantly greater in Zol vs. VC and Vab. (**F**) Trabecular separation (Tb.Sp) is significantly smaller in Zol, Vab and Zol/Vab vs. VC, and in Zol and Zol/Vab vs. Vab. (**G**) Bone mineral density (BMD) is significantly greater in Zol and Zol/Vab vs. VC and Vab, although BMD is the same in Vab and VC. (**H**) The number of osteoclasts on the bone surface (N.Oc/BS) is significantly decreased in Zol, Vab, Zol/Vab vs. VC. (**I**) Representative hematoxylin and eosin-stained and immunofluorescent images of tongue tissue (white arrowheads: CD31^+^ cells, bar: 100 μm). (**J**) The number of CD31^+^ blood vessels (CD31^+^ BVs) is significantly decreased in Zol, Vab, and Zol/Vab vs. VC. Graphs show means ± SEM. * *p* < 0.05, ** *p* < 0.01, *** *p* < 0.001, † *p* < 0.05, †† *p* < 0.01, ††† *p* < 0.001; *n* = 5 mice/group.

As expected, Zol administration significantly decreased serum TRAcP5b levels, regardless of Vab administration, when compared to VC (Figure 2A). Moreover, administration of Vab significantly decreased the serum level of TRAcP5b compared to VC (Figure 2A). Zol administration significantly increased BV/TV, with increases in Tb.N, Tb.Th, and BMD, and decreases in Tb.Sp, irrespective of Vab administration, compared to VC (Figure 2B–G). Vab administration also significantly increased BV/TV with a decrease in Tb.Sp compared to VC (Figure 2B,C,F). Zol administration significantly decreased N.Oc/BS compared to VC, irrespective of Vab administration, whereas Vab administration also significantly decreased N.Oc/BS compared to VC with no synergistic effect of the Zol/Vab combination (Figure 2H). On the other hand, administration of both Zol and Vab significantly decreased the number of CD31^+^ blood vessels in the tongue connective tissues (Figure 2I,J).

### 3.2. Effects of Drugs on Gross Wound and Bone Architecture of Tooth Extraction Sockets

**Figure 3:**
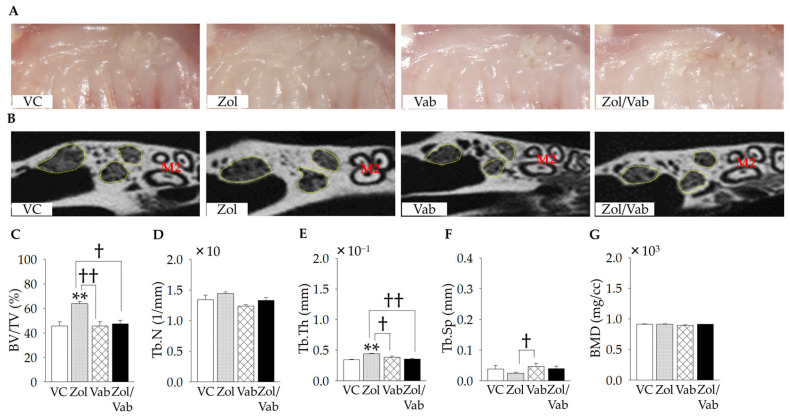
Effects of administered drugs on gross healing and bone architecture of tooth extraction sockets. (**A**) Representative intra-oral photos in the occlusal view. No open wounds are noted in all groups. (**B**) Representative micro computed tomography images of tooth extraction sockets (yellow dotted surrounded areas: mesial and distal roots of first molars, M2: second molars). (**C**) Bone fill of tooth extraction sockets per tissue volume (BV/TV) is significantly higher in Zol vs. other comparison groups. (**D**) Trabecular number (Tb.N) is similar among the groups. (**E**) Trabecular thickness (Tb.Th) is significantly greater in Zol vs. other comparison groups. (**F**) Trabecular separation (Tb.Sp) is significantly smaller in Zol vs. Vab. (**G**) Bone mineral density (BMD) is similar in all groups. Graphs show means ± SEM. ** *p* < 0.01, † *p* < 0.05, †† *p* < 0.01; *n* = 5 mice/group.

Gross wound healing and bone architecture of tooth extraction sockets were examined. Gross healing of tooth extraction sockets appeared to be complete in all experimental groups (Figure 3A). Indeed, no open wounds around tooth extraction sockets were identified based on intra-oral photos and H&E-stained sections. Zol administration significantly increased bone fill of tooth extraction sockets (BV/TV) compared to all comparison groups (Figure 3B,C). Only Tb.Th and Tb.Sp were significantly increased and decreased, respectively, with Zol administration compared to Vab administration (Figure 3B–G).

### 3.3. Effects of Drugs on Osseous Wound Healing of Tooth Extraction Sockets

**Figure 4:**
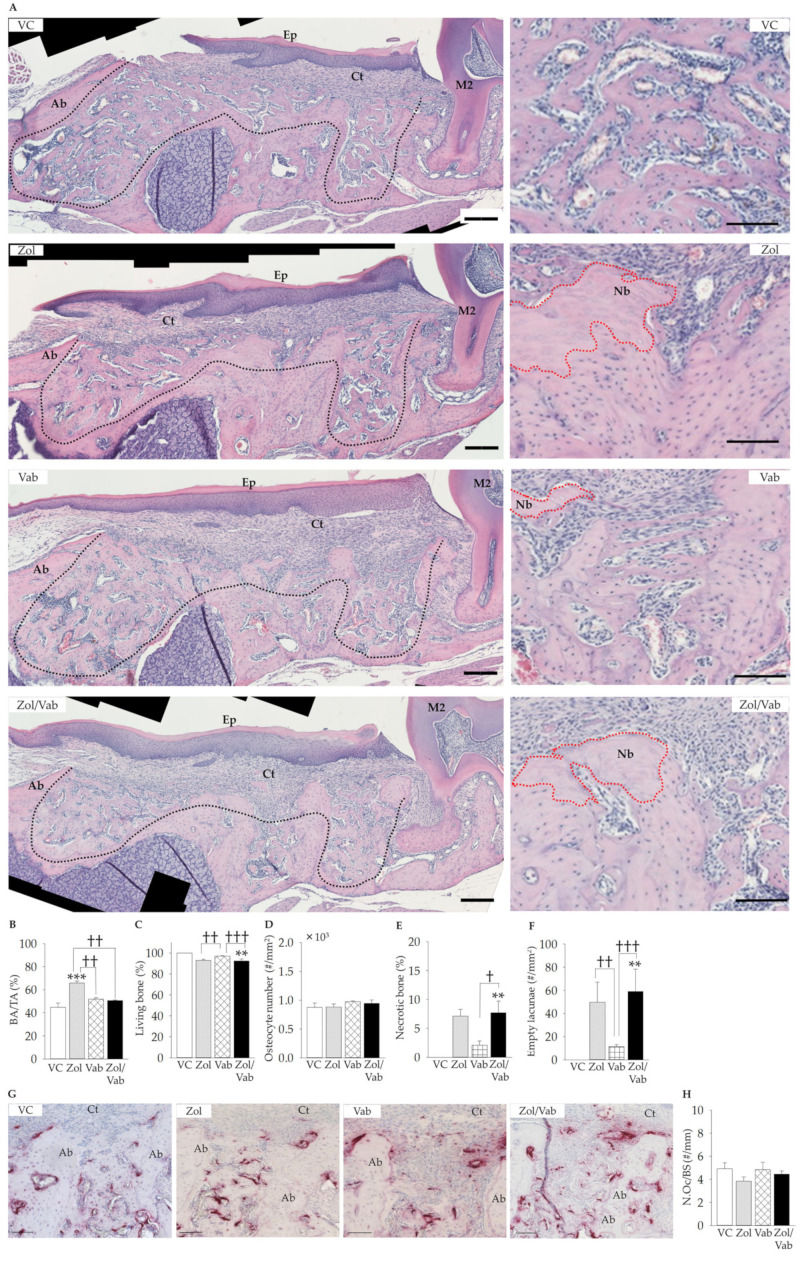
Effects of administered drugs on osseous wound healing of tooth extraction sockets. (**A**) Representative hematoxylin and eosin-stained sagittal images of tooth extraction sockets [small (left) and higher (right) magnification, black and red dotted line: tooth extraction sockets and necrotic bone, respectively, bar: 200 μm]. (**B**) Bone area per tissue area (BA/TA) in the tooth extraction sockets is significantly increased in Zol vs. other comparison groups. (**C**) Living bone area (living bone) is significantly decreased in Zol/Vab vs. VC and Vab. (**D**) The distribution of osteocytes in the bone tissue (osteocyte number) is almost the same among the groups. (**E**) Necrotic bone area (necrotic bone) is significantly increased in Zol/Vab vs. VC and Vab, whereas it is increased in Zol vs. VC, but not significantly. (**F**) The distribution of empty lacunae (empty lacunae) in bone tissue is significantly increased in Zol/Vab vs. VC and Vab, and in Zol vs. Vab, whereas it is increased in Zol vs. VC, but not significantly. (**G**) Representative TRAP-stained images of tooth extraction sockets (Ab: alveolar bone, CT: connective tissue, bar: 100 μm). (**H**) The number of osteoclasts on the bone surface in the tooth extraction sockets is similar among the groups. Graphs show means ± SEM. ** *p* < 0.01, *** *p* < 0.001, † *p* < 0.05, †† *p* < 0.01, ††† *p* < 0.001; *n* = 5 mice/group.

Osseous healing appeared to be similar among the groups. However, histological features were quite different under higher magnifications. Zol administration significantly increased bone fill of tooth extraction sockets compared to all comparison groups (Figure 4A,B). The Zol/Vab combination significantly decreased living bone and increased necrotic bone, with an increase in the number of empty lacunae compared to VC and Vab, whereas similar findings were noted with Zol administration, but they were not significant (Figure 4B–F). No difference was observed in N.Oc/BS in the tooth extraction sockets in all groups (Figure 4G,H).

Therefore, Zol administration worsened osseous wound healing of tooth extraction sockets, which was accelerated by the Zol/Vab combination from the perspective of living bone, necrotic bone, and the number of empty lacunae.

### 3.4. Effects of Drugs on Soft Tissue Healing of Tooth Extraction Sockets

**Figure 5:**
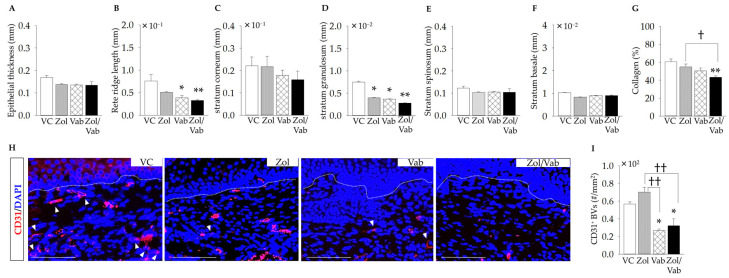
Effects of administered drugs on soft tissue wound healing of tooth extraction sockets. (**A**) No change is observed in epithelial thickness. (**B**) Rete ridge length is significantly decreased in Zol/Vab and Vab vs. VC. (**C**) Thickness of the stratum corneum is the same among the groups. (**D**) Thickness of the stratum granulosum is significantly decreased in Zol, Vab, and Zol/Vab vs. VC. (**E**,**F**) Thicknesses of the stratum spinosum and stratum basale are almost the same among the groups. (**G**) Collagen production is significantly decreased in Zol/Vab vs. VC and Zol. (**H**) Representative immunofluorescent images of CD31^+^ blood vessels (BVs) in the connective tissue of tooth extraction sockets (white arrowheads: CD31^+^ cells, bar: 100 μm). (**I**) The number of CD31^+^ BVs is significantly decreased in Vab and Zol/Vab vs. VC and Zol. Graphs show means ± SEM. * *p* < 0.05, ** *p* < 0.01, † *p* < 0.05, †† *p* < 0.01; *n* = 5 mice/group.

Soft tissue healing appeared to be similar in all groups without bone exposure, based on intra-oral images and H&E staining under lower magnification (Figure 3A and Figure 4A). However, quantitative histological analyses of epithelium under higher magnification showed some differences. The Zol/Vab combination and Vab alone significantly reduced rete ridge length and the thickness of the stratum granulosum compared to VC, although they did not affect the thicknesses of the stratum corneum, stratum spinosum, and stratum basale in all groups (Figure 5A–F). Zol, Vab, and the Zol/Vab combination tended to decrease epithelial thickness (Figure 5A). Moreover, the Zol/Vab combination significantly reduced collagen production in the connective tissue of tooth extraction sockets compared to VC and Zol alone (Figure 5G). Next, the distribution of blood vessels was investigated. Vab significantly reduced the distribution of blood vessels in the connective tissue of tooth extraction sockets, regardless of its combination with Zol, compared to the VC and Zol groups (Figure 5H,I).

Therefore, Zol/Vab induced abnormal epithelial healing and exaggerated connective tissue healing of tooth extraction sockets.

### 3.5. Effects of Drugs on Macrophages in the Tooth Extraction Sockets

**Figure 6:**
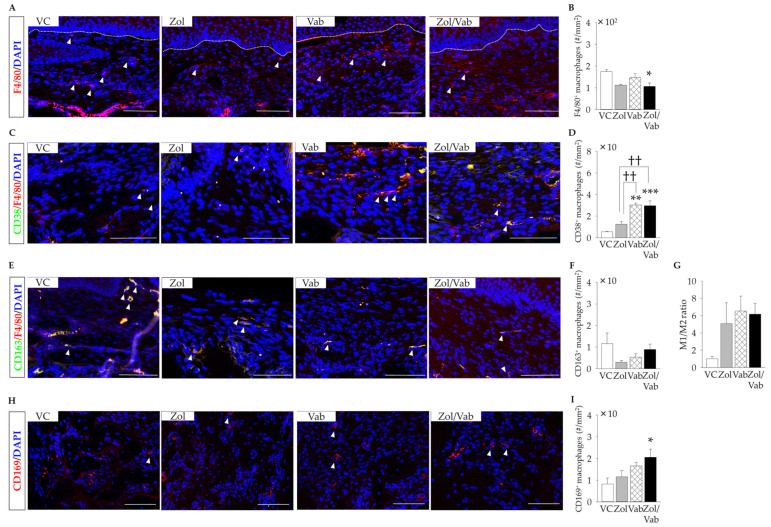
Effects of administered drugs on macrophages in the tooth extraction sockets. (**A**) Representative fluorescent image of F4/80^+^ macrophages (white arrowheads: F4/80^+^ macrophages, bar: 100 μm). (**B**) The number of F4/80^+^ macrophages is significantly decreased in Zol/Vab vs. other comparison groups. (**C**) Representative fluorescent images of CD38^+^ macrophages (white arrowheads: CD38^+^ macrophages, bar: 100 μm). (**D**) The number of CD38^+^ macrophages is significantly increased in Zol/Vab vs. other comparison groups. (**E**) Representative fluorescent images of CD163^+^ macrophages (white arrowheads: CD163^+^ macrophages, bar: 100 μm). (**F**) The number of CD163^+^ macrophages is similar among the comparison groups. (**G**) The M1/M2 ratio is slightly higher in Vab and Zol/Vab vs. VC. (**H**) Representative fluorescent images of CD169^+^ macrophages (osteomacs) (white arrowheads: CD169^+^ macrophages, bar: 100 μm). (**I**) The number of CD169^+^ macrophages is greater in Zol/Vab vs. other comparison groups. Graphs show means ± SEM. * *p* < 0.05, ** *p* < 0.01, *** *p* < 0.001, †† *p* < 0.01; *n* = 5 mice/group.

Finally, the distribution of macrophages in soft tissue and bone marrow tissue was examined. The Zol/Vab combination significantly reduced the number of F4/80^+^ macrophages in the connective tissue of tooth extraction sockets compared to VC (Figure 6A,B). Zol administration also tended to decrease the number of F4/80^+^ macrophages, but not significantly (Figure 6A,B). Vab significantly increased the number of F4/80^+^CD38^+^ macrophages in the connective tissue of tooth extraction sockets, irrespective of Zol administration, compared to the VC and Zol groups (Figure 6C,D). On the other hand, the distribution of F4/80^+^CD163^+^ macrophages was the same in the connective tissue of tooth extraction sockets in all comparison groups (Figure 6E,F), which resulted in an increased ratio of M1 to M2 macrophages in the Zol, Vab and Zol/Vab groups, but not significantly (Figure 6G). Interestingly, only the Zol/Vab combination, but not Zol and Vab alone, significantly increased the number of CD169^+^ osteal macrophages in the tooth extractions sockets compared with VC (Figure 6H,I). Zol and Vab alone did not affect the number of CD169^+^ osteal macrophages in the tooth extraction sockets, as with VC administration (Figure 6H,I).

## 4. Discussion

A murine model of MRONJ Stage 0-like lesions without bone exposure was induced by the Zol/Vab combination with tooth extraction, with increased necrotic bone and empty lacunae, abnormal epithelial healing due to reduced rete ridge length and thickness of the stratum granulosum, and delayed connective tissue healing due to suppressed collagen production. We first demonstrated that the Zol/Vab combination resulted in significant accumulation of CD169^+^ osteal macrophages in the bone marrow in MRONJ Stage 0-like lesions with significant suppression of F4/80^+^ macrophage distribution, but increases in CD38^+^ macrophages in the connective tissue. Moreover, we found that necrotic bone formation was induced first, regardless of soft tissue wound dehiscence in the tooth extraction sockets affected by the Zol/Vab combination.

Anti-VEGFA monoclonal antibody suppresses proliferation and survival of vascular endothelial cells by inhibition of binding vascular endothelial growth factor A (VEGFA) to the receptor of VEGF [27], whereas anti-VEGFA monoclonal antibody has been shown to suppress bone resorption by inhibiting differentiation and survival of osteoclasts via blocking the VEGF-VEGFR1 pathway in osteoclasts [28,29,30], in accordance with the increased bone volume with decreases in the serum TRAcP5b level and the reduced number of osteoclasts in femora in the present study. Therefore, anti-VEGFA monoclonal antibody negatively affected bone remodeling in systemic bones. Bevacizumab is clinically used at a dosage of 5 to 10 mg/kg every 2 or 3 weeks in the treatment of patients with malignancies with chemotherapy. The dosage of Vab used in the present study was about half the dose for clinical use, based on the formula for determining dosage in animals [31], although the dosage of Zol used in the present study was almost the same as for clinical use [16,22,25]. Histopathological and immunopathological findings in the tooth extraction sockets were significantly worsened with about half the dosage for clinical use compared to one-third of the dosage in our preliminary study. Thus, about a half but not the same dosage of Vab for clinical use was used in combination with Zol to create a murine model of MRONJ Stage 0-like lesions without bone exposure. According to the ages matching formula of laboratory mice and humans, the mice used in this study (tooth extraction at 11-week-old and euthanasia at 13-week-old) is are approximately equal to 30–35 years old in humans [32], which suggests that the healing period of extraction sockets is different or shortened compared to older mice [33,34]. Therefore, not only Zol and Vab dosages but also the murine age used in this study could be associated with complete epithelial coverage of tooth extraction sockets.

Bevacizumab or inhibition of angiogenesis is one of the high-risk factors for developing MRONJ [3]. However, whether inhibition of angiogenesis is the main cause for developing MRONJ remains controversial [6,35,36,37,38,39]. No difference or reduction of the distribution of blood vessels in or around BRONJ lesions was noted based on histological findings compared to osteoradionecrosis or healthy samples in humans [36,37]. Angiogenesis-related genes obtained from peripheral blood mononuclear cells were not found to differ between multiple myeloma patients with BRONJ and healthy volunteers [38]. The present findings based on histopathological analyses were partially accounted for in our previous study investigating the effects of anti-VEGFA neutralizing antibody on tooth extraction socket healing [23]. Therefore, the current and previous findings strongly suggest that anti-VEGFA antibody therapy is one of the risk factors for, but not the main cause of, development of MRONJ, since angiogenesis inhibitor monotherapy was excluded as a causative drug in the latest AAOMS position paper [3].

Interestingly, wound healing of both soft and hard tissues of tooth extraction sockets was significantly worsened or abnormal when Vab was administered in combination with Zol, although gross wound healing appeared normal. To the best of our knowledge, clinically, the histological findings of MRONJ Stage 0 have never been demonstrated due to the difficulty of diagnosis of similar gross healing to normal healing and ethical issues, although there are clinical symptoms and radiographic findings [3,20]. However, in the present study using the Vab/Zol combination with tooth extraction, hard tissue of the tooth extraction socket had necrotic bone areas without wound exposure, which strongly suggests that necrotic bone potentially develops with no correlation to bacterial infections. There may also be necrotic bone in MRONJ Stage 0 in clinical situations as in the findings of the present study, although the existence of necrotic bone has not been listed in the current position papers [3,18,19,20].

It is unclear why open wounds develop in MRONJ. In keratinized oral mucosa, the epithelium is comprised of 4 layers, as follows: the stratum basale with rete ridge, stratum spinosum, stratum granulosum, and stratum corneum (parakeratin layer). In the present study, rete ridge length and the thickness of the stratum granulosum were significantly abnormal with epithelial coverage with Vab administration, regardless of its combination with Zol administration. The rete ridge, which is composed of the stratum spinosum and stratum basale [40], is the gingival epithelium elongating into connective tissue. It has been demonstrated that decreased rete ridge length significantly induced detachment of the epithelial layer from connective tissue [41]. The tight junction system, which functions to seal cells together, to maintain cell polarity, to create a permeability barrier to water, and to enhance cell adhesion, has been demonstrated to be predominantly localized in the stratum granulosum [42,43,44]. Therefore, reduced barrier systems due to decreased thickness of the stratum granulosum in gingival epithelium in the present study may be associated with the progression to higher stages of MRONJ from MRONJ stage 0 without bone exposure.

In our previous study investigating the effects of chemotherapy drugs and Zol combination administration on soft and hard tissue wound healing of tooth extraction sockets, the distribution of F4/80^+^ macrophages was significantly decreased in the connective tissue of MRONJ-like lesions [16]. Our other previous studies also demonstrated that resolution of MRONJ-like lesions by transplantation of adipose-derived stromal vascular fraction cells and quantity and quality controlled peripheral mononuclear cells significantly shifted subset polarization of macrophages to M2 macrophages [24,45]. Another recent clinical investigation reported that polarization shifting of macrophage subsets (M1 macrophages: inflammatory macrophages vs. M2 macrophages: anti-inflammatory macrophages) was different between MRONJ Stage 1 and Stage 2 or 3. Therefore, it was strongly suggested that polarization shifting of macrophage subsets by decreased distribution of F4/80^+^ macrophages, but a slightly increased ratio of M1 macrophages, in the connective tissue of tooth extraction sockets induced by the Vab/Zol combination is associated with the immunopathology of the early stages of MRONJ (Stage 0).

To the best of our knowledge, there have been no studies reporting the relationship between osteal macrophages and MRONJ. Osteal macrophages account for approximately 15% to 20% of total bone marrow cells in murine long bones [8], although there is no evidence regarding the ratio in the bone marrow of jawbones. Osteal macrophages have been demonstrated to be required for and/or enhance both intramembranous and endochondral long bone healing after injury by supporting osteoblasts [10,12]. Moreover, depletion of osteal macrophages has also been shown to abolish or delay long bone healing [10,12], strongly suggesting that osteal macrophages are imperative for bone formation during bone repair or bone anabolism, although the effects of osteal macrophages on osseous wound healing of tooth extraction are unclear. On the other hand, more recently, it has been shown that osteal macrophages strongly support bone resorption with osteoclasts by phagocytosis and sequestration of post-bone resorption byproducts. It has been shown that osteal macrophages contribute to pathogenesis of one of the bone diseases, osteoporosis [46]. Moreover, osteal macrophages can differentiate into osteoclasts [47]. Therefore, differentiation inhibition of osteal macrophages into osteoclasts induced by Vab and Zol combination resulted in accumulation of osteal macrophages in bone marrow, which may result in the pathogenesis or pathophysiology of MRONJ Stage 0, as well as the pathogenesis of osteoporosis [46], although the exact mechanism is unclear. More polarization shifting of macrophage subsets such as M1, M2, and osteal macrophages may become one of the determining factors for progression to a higher stage of MRONJ from MRONJ Stage 0. However, it has been demonstrated that aging strongly affects the function and/or polarization of macrophages in several types of tissues such as skeletal muscle [48], enteric nervous system [49], bone marrow of long bone [50], injured liver [51], and spleen [52]. The mice used in this study were relatively young. Therefore, caution should be taken when considering the alteration of macrophage population in this study with clinical situations, since the clinical chance of combination use of Vab and Zol in young adult women is relatively low.

## 5. Conclusions

Within the limitations of this study due to the experimental time schedule, dosage of administered drugs, and its being an animal study, to our knowledge, we were the first to create a murine model of MRONJ Stage 0-like lesions induced by Zol and anti-VEGFA neutralizing antibody with tooth extraction. We further demonstrated that accumulation of osteal macrophages in the bone marrow of the jawbone and decreased macrophages with a slightly increased ratio of M1 macrophages in the connective tissue are involved in the immunopathology of MRONJ Stage 0-like lesions in mice.

## Data Availability

The data supporting the findings of this study are available within the article.
